# Chest computed tomography in COVID-19 pneumonia: a retrospective study of 155 patients at a university hospital in Rio de Janeiro, Brazil

**DOI:** 10.1590/0100-3984.2020.0133

**Published:** 2021

**Authors:** Roberto Mogami, Agnaldo Jose Lopes, Ronaldo Carvalho Araújo Filho, Fernando Carlos Santos de Almeida, Alexandre Malta da Costa Messeder, Ana Celia Baptista Koifman, Amanda Barbosa Guimarães, Alexandra Monteiro

**Affiliations:** 1 Hospital Universitário Pedro Ernesto da Universidade do Estado do Rio de Janeiro (UERJ), Rio de Janeiro, RJ, Brazil.

**Keywords:** Coronavirus infections/diagnostic imaging, Tomography, X-ray computed, Pneumonia, viral, Infecções por coronavírus/diagnóstico por imagem, Tomografia computadorizada por raios X, Pneumonia viral

## Abstract

**Objective:**

To define diagnostic criteria for coronavirus disease 2019 (COVID-19) on computed tomography (CT); to study the correlation between CT and polymerase chain reaction (PCR) testing for infection with severe acute respiratory syndrome coronavirus 2; and to determine whether the extent of parenchymal involvement and the need for mechanical ventilation are associated with the CT findings and clinical characteristics of patients with COVID-19.

**Materials and Methods:**

This was a retrospective study of 155 patients with COVID-19 treated between March and May 2020. We attempted to determine whether the CT findings correlated with age and clinical variables, as well as whether the need for mechanical ventilation correlated with the extent of the pulmonary involvement.

**Results:**

On average, the patients with COVID-19 were older than were those without (mean age, 54.8 years vs. 45.5 years; *p* = 0.031). The most common CT finding (seen in 88.6%) was ground-glass opacity, which correlated significantly with a diagnosis of COVID-19 (*p* = 0.0001). The CT findings that correlated most strongly with the need for mechanical ventilation were parenchymal bands (*p* = 0.013), bronchial ectasia (*p* = 0.046), and peribronchovascular consolidations (*p* = 0.012). The presence of one or more comorbidities correlated significantly with more extensive parenchymal involvement (*p* = 0.023). For the diagnosis of COVID-19, CT had a sensitivity of 84.3%, a specificity of 36.7%, and an accuracy of 73.5% (*p* = 0.012 vs. PCR).

**Conclusion:**

The patterns of CT findings are useful for the diagnosis of COVID-19 and the evaluation of disease severity criteria. The presence of any comorbidity is associated with greater severity of COVID-19.

## Abstract (pt)

**Objetivo:**

Definir os critérios diagnósticos da COVID-19 na tomografia computadorizada (TC), estudar a concordância entre a TC e o PCR e determinar a associação da extensão do envolvimento parenquimatoso e ventilação mecânica com os achados tomográficos e características clínicas da amostra.

**Materiais e Métodos:**

Estudo de 155 pacientes com COVID-19 atendidos entre março e maio de 2020. Tentamos determinar se os achados da TC se correlacionavam com a idade e variáveis clínicas, bem como se a necessidade de ventilação mecânica se correlacionava com a extensão do envolvimento pulmonar.

**Resultados:**

A faixa etária dos pacientes com COVID-19 (54,8 anos) foi maior do que a dos pacientes sem a doença (45,5 anos) (*p* = 0,031). Opacidades em vidro fosco foram as alterações mais frequentes (88,6%; *p* = 0,0001). Bandas parenquimatosas (*p* = 0,013), ectasia brônquica (*p* = 0,046) e consolidações peribroncovasculares (*p* = 0,012) foram mais frequentes nos pacientes que necessitaram de ventilação mecânica. Pacientes com comorbidades apresentaram envolvimento parenquimatoso mais extenso (*p* = 0,023). A TC apresentou sensibilidade de 84,3%, especificidade de 36,7% e acurácia de 73,5% (*p* = 0,012).

**Conclusão:**

Os padrões de TC são úteis para o diagnóstico de COVID-19 e avaliação dos critérios de gravidade da doença. Pacientes com comorbidades estão associados a condições clínicas mais graves.

## INTRODUCTION

Imaging methods have played a prominent role in the diagnosis of coronavirus disease 2019 (COVID-19) and in the follow-up of patients with the disease^(1-9)^. Chest computed tomography (CT) has always been the protagonist of this process, and various radiological societies were efficient in disseminating consensus statements about the use of imaging methods for the diagnosis of COVID-19^(10,11)^. Although polymerase chain reaction (PCR) is the gold standard for the diagnosis of infection with severe acute respiratory syndrome coronavirus 2 (SARS-CoV-2), CT is an alternative for situations in which it is necessary to make immediate decisions^(12)^. However, to our knowledge, there have been few studies using objective diagnostic criteria to evaluate the accuracy of CT in the diagnosis of COVID-19, as well as analyzing interobserver agreement.

The objectives of this study were to characterize the clinical and CT findings of a sample of SARS-CoV-2-positive patients admitted to the hospital with COVID-19 pneumonia; to define diagnostic CT criteria for COVID-19, evaluating the correlation between CT and PCR, as well as the interobserver agreement for chest CT scans; and to determine whether the associations of the extent of pulmonary parenchymal involvement on CT and the need for mechanical ventilation correlate with CT findings, clinical variables, and epidemiological characteristics.

## MATERIALS AND METHODS

The study was approved by the Institutional Review Board of Pedro Ernesto University Hospital, operated by Rio de Janeiro State University, in the city of Rio de Janeiro, Brazil (Reference no. 31363230.1.0000.5282). Because all of the data evaluated were obtained retrospectively from the database of the hospital, the requirement for written informed consent was waived.

### Subjects

We selected 155 patients with suspected COVID-19 treated at Pedro Ernesto University Hospital between March and May 2020. All of the patients underwent PCR tests. We excluded 23 patients in whom the data were insufficient for a complete analysis of the associations. Therefore, the final sample comprised 132 patients. However, to determine the observer accuracy for CT patterns and the level of interobserver agreement, we used the CT scans and PCR test results for all 155 patients.

The inclusion criteria were being ≥ 18 years of age, presenting with acute respiratory symptoms, and having had contact with COVID-19 patients or individuals with symptoms suggestive of the disease, with or without laboratory confirmation of SARS-CoV-2 infection. Patients in whom the technical standards of the CT scans were deemed unacceptable (movement artifacts or missing sequences) were excluded, as were those for whom clinical or epidemiological data were missing.

### Chest CT analysis

Three radiologists, each with more than 20 years of experience, independently analyzed the CT patterns and categorized disease probability using a classification system adapted from the Radiological Society of North America (RSNA) consensus^(13)^. We divided the CT aspects into four patterns: typical, possible, and atypical of viral disease (including COVID-19); and negative for lung disease. The findings that characterized those patterns were as follows:

- typical-ground-glass opacities (peripheral, bilateral, rounded, or multifocal) or the reversed halo sign; with or without consolidations; and with or without the crazy-paving pattern
- possible-the absence of the typical appearance; and unilobular, perihilar, non-peripheral, non-rounded ground-glass opacities
- atypical-the absence of the typical and possible patterns; and the presence of lobar/segmental cavitation, consolidation, micronodules, bronchiolar opacities, or smooth septal thickening, with pleural effusion or masses
- negative for lung disease-no findings indicative of pulmonary alterations

Because there was no disagreement between the readers for the negative lung disease pattern, we calculated the kappa statistic (to quantify interobserver agreement) only for the three other patterns. The typical and possible patterns were both considered indicative of positivity for COVID-19, and, in accordance with the clinical practice protocol at our institution, patients presenting with the possible pattern were considered potential carriers of the disease and were therefore isolated. The patients were also evaluated according to the classification system devised by Pan et al.^(14)^, which estimates the stage of disease progression (days after symptom onset) on the basis of the CT findings: the early stage, or stage 1 (0-4 days); the progressive stage, or stage 2 (5-8 days); the peak stage, or stage 3 (9-13 days); and the absorption stage, or stage 4 (≥ 14 days). In addition, we assessed the proportion of lung parenchyma involved, as visualized in three orthogonal planes (axial, coronal, and sagittal), a standard procedure performed at many institutions, categorizing the involvement as < 25%, 25-50%, or > 50% of the total area. The proportional involvement of the lung parenchyma was also evaluated as a dichotomous variable (≤ 50% or > 50%).

### Statistical analysis

Continuous variables were compared by Student’s t-test for independent samples or the Mann-Whitney test, as appropriate, whereas categorical variables were compared by the chi-square test or Fisher’s exact test. The distribution of the data was evaluated by the Shapiro-Wilk test and in histograms. The time from the onset of symptoms to CT did not show a normal (Gaussian) distribution. Therefore, the adequate measures to summarize these data were median and interquartile range. The level of significance was set at 5%. The statistical analysis was performed with the SPSS Statistics software package, version 26 (IBM Corp., Armonk, NY, USA).

## RESULTS

### Sample characteristics

Of the 132 patients evaluated, 72 (54.5%) were male and 60 (45.5%) were female. The mean age of the patients was 52.9 ± 16.1 years. Ninety-seven of the patients (73.5%) had been referred from screening centers, and the remaining 35 patients (26.5%) were inpatients at the hospital.

### Clinical findings

The time from the onset of symptoms to CT was 1-4 days in 41 patients (31.1%), 5-8 days in 47 (35.6%), 9-13 days in 21 (15.9%), and ≥ 14 days in 23 (17.4%). Of the 132 patients evaluated, 61 (46.2%) had one or more comorbidities and 71 (53.8%) had no comorbidities. The main comorbidities were as follows: arterial hypertension, in 31 patients; diabetes mellitus, in 18 patients; obesity, in nine patients; respiratory diseases (mainly asthma and chronic obstructive pulmonary disease), in eight patients; cardiac diseases (cardiac insufficiency or heart valve disease), in six patients; malignant neoplasms, in five patients; renal diseases (lithiasis or chronic kidney disease), in four patients; and hematological diseases (sickle cell disease or thalassemia), in four patients; and rheumatological diseases (gout and lupus), in two patients. In addition, 12 patients (9.1%) required mechanical ventilation and 120 (90.9%) did not.

### CT findings

Among the 155 patients in the overall sample, the chest CT pattern, vis-à-vis COVID-19 pneumonia, was classified as typical in 86 (65.2%), possible in 19 (19%), and atypical in 14 (10.6%), whereas the chest CT pattern was classified as negative for lung disease in 13 patients (9.8%). There were 105 patients in whom the chest CT findings were diagnostic of COVID-19 (CT-positive group) and 27 in whom the chest CT showed no signs of COVID-19 (CT-negative group). Among those patients, the disease progression was categorized as stage 1 in 32 (30.5%), stage 2 in 41 (39.0%), stage 3 in 30 (28.6%), and stage 4 in only two (1.9%).

The diagnosis of COVID-19 was not significantly associated with gender or patient origin (outpatient or inpatient). The mean age was 54.8 ± 14.5 years among the patients with COVID-19, compared with 45.5 ± 20.1 years among those without, and the difference was statistically significant (*p* = 0.031). In the CT-positive group, the time from the onset of symptoms to CT was 1-4 days in 27 (25.7%) of the patients, 5-8 days in 43 (41.0%), 9-13 days in 18 (17.1%), and ≥ 14 days in 17 (16.2%). In the CT-negative group, the time from the onset of symptoms to CT was 1-4 days in 14 (51.9%) of the patients, 5-8 days in 4 (14.8%), 9-13 days in 3 (11.1%), and ≥ 14 days in 6 (22.2%). For that variable, the difference between the two groups was significant (*p* = 0.019). Among the patients in the CT-positive group, the proportion of the lung parenchyma involved was < 25% in 37 (35.2%), 25-50% in 22 (21.0%), and > 50% in 46 (43.8%).

**Figure 1:**
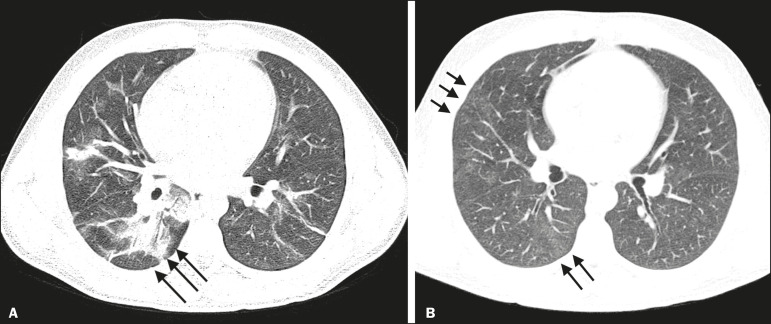
A 29-year-old male patient. **A**: CT scan acquired on day 4 after symptom onset, showing peribronchovascular consolidations (a stage 3 finding) in the right lower lobe (arrows). **B**: CT scan acquired on day 14 after symptom onset. The patient still had a mild cough and dyspnea. There are discrete residual ground-glass opacities (a stage 4 finding) in the right lung (arrows).

**Figure 2:**
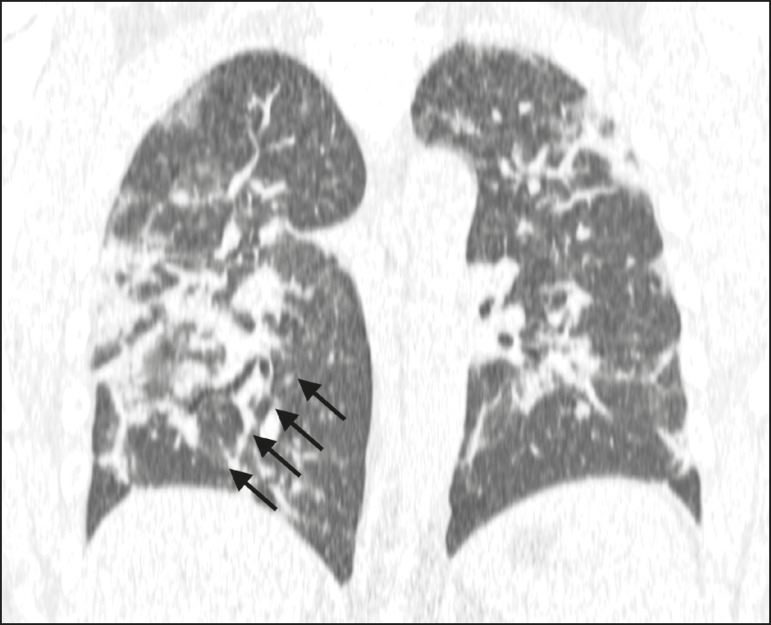
A 36-year-old male patient at 15 days after symptom onset. Chest CT scan showing areas of consolidation together with bronchial ectasia (arrows).

**Figure 3:**
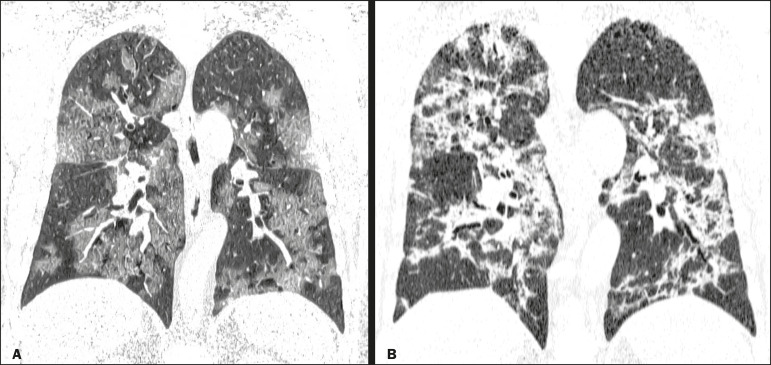
A 48-year-old male patient at 10 days after symptom onset. **A**: CT reconstruction in the coronal plane, showing a diffuse crazy-paving pattern (a stage 2 finding). **B**: At 21 days after symptom onset, this patient was admitted to the intensive care unit with a diffuse pattern of consolidations due to organizing pneumonia (a stage 3 finding).

**Figure 4:**
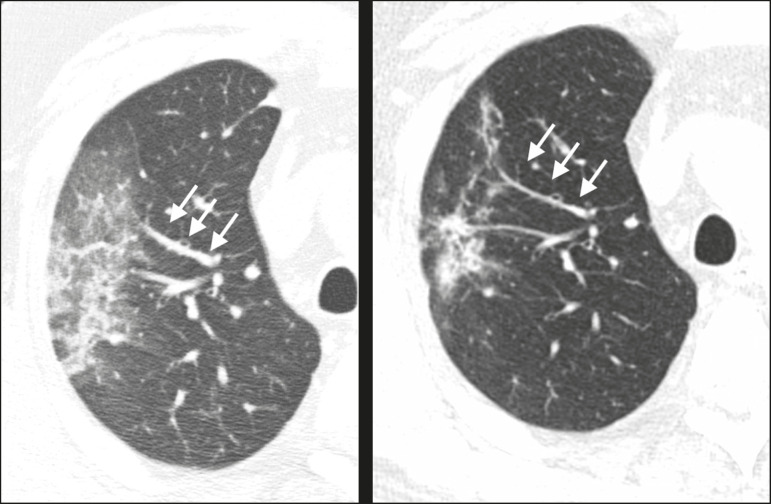
A 64-year-old male patient. **A**: CT scan acquired on day 3 after symptom onset. Note the increased vessel caliber in the parenchyma of the right upper lobe (arrows), adjacent to an area with the crazy-paving pattern. **B**: CT scan, acquired 28 days after the first CT, showing reduced vessel caliber and resolution of the crazy-paving pattern, with small residual consolidation.

Among the 105 patients in the CT-positive group, the chest CT showed ground-glass opacities, as depicted in Figure 1, in 93 (88.6%), and that finding was significantly associated with the diagnosis (*p* < 0.0001). Other chest CT findings significantly associated with the diagnosis of COVID-19 included conventional consolidation (Figures 2 and 3), observed in 45 (42.9%) of the patients (*p* = 0.020); the crazy-paving pattern (Figure 3), in 44 (41.9%; *p* = 0.0008); parenchymal bands, in 34 (32.4; *p* = 0.009); vascular thickening (Figure 4), in 24 (22.9%; *p* = 0.002); peribronchovascular consolidation (Figures 1 and 3), in 23 (21.9%; *p* = 0.003); nodules or consolidations with the halo sign, in 15 (14.3%; *p* = 0.025); subpleural lines, in 15 (14.3%; *p* = 0.025). The chest CT findings that were not significantly associated with the diagnosis of COVID-19 included bronchial ectasia (Figure 2), in 12 patients (11.4%; *p* = 0.21); pleural effusion, in ten (9.5%; *p* = 0.31); architectural distortion, in seven (6.7%; *p* = 0.19); the bubble sign, in three (2.9%; *p* = 0.50); and the reversed halo sign, in one (0.8%; *p* = 0.80).

In the CT-positive group, the extent of parenchymal involvement was significantly associated with the need for mechanical ventilation (*p* = 0.023), the stage of disease progression (*p* < 0.0001), and the time from the onset of symptoms to CT (*p* = 0.011). We also observed an association between the presence of any comorbidity and the need for mechanical ventilation (*p* = 0.023). The chest CT findings that were significantly associated with the need for mechanical ventilation were parenchymal bands (*p* = 0.013), bronchial ectasia (*p* = 0.046), and peribronchovascular consolidations (*p* = 0.012). The extent of parenchymal involvement was also significantly associated with conventional consolidation (*p* = 0.003), peribronchovascular consolidation (*p* = 0.019), nodule/consolidation with the halo sign (*p* = 0.001), and pleural effusion (*p* = 0.017).

### Agreement between CT and PCR for the diagnosis of COVID-19

For the diagnosis of COVID-19, CT was found to have a sensitivity of 84.3%, a specificity of 36.7%, a positive predictive value of 81.9%, a negative predictive value of 40.7%, and an overall accuracy of 73.5%, with positive and negative likelihood ratios of 1.33 and 0.43, respectively). There was significant agreement between CT and PCR for the diagnosis of COVID-19 in the sample (kappa = 0.22; 95% CI: 0.0-0.44; *p* = 0.012).

### Interobserver agreement

The level of agreement among the three observers was moderate but significant (*p* < 0.001). The agreement was best between observers one and two (kappa = 0.54; standard error: 0.07; *p* < 0.001), with a concordance rate of 76.8%.

## DISCUSSION

Our study showed the importance of CT patterns in the diagnosis of COVID-19 and in the evaluation of some disease severity criteria. The CT patterns were concordant with the PCR results, and there was also moderate agreement among the three CT readers. There was an association between various CT findings with the clinical findings, the need for mechanical ventilation, and the extent of pulmonary involvement.

Gender did not influence the diagnosis of COVID-19, the need for mechanical ventilation, or the extent of parenchymal involvement. On average, the patients with COVID-19 were older (mean age, 54.8 years) than were those without (mean age, 45.5 years). In addition, the patients diagnosed with COVID-19 showed symptom onset predominantly at 5-13 days. It is possible that, between days 1 and 4, the findings were too incipient or atypical to be diagnostic, and that, after day 14, there was regression of the inflammatory process.

Barbosa et al.^(15)^ found that, when typical and indeterminate findings were considered as positive, chest CT showed an accuracy of 70.3% for the diagnosis of COVID-19, lower than the 73.5% obtained in the present study, although the sensitivity and specificity reported by those authors were both higher than that seen in our sample (92.0% and 62.1% vs. 84.3% and 36.7%, respectively). There was concordance between our reading patterns and PCR. To our knowledge, this was the first study to evaluate such concordance. The sensitivity of CT identified in our study was also lower than that found in several other studies^(12,16,17)^, although none of those studies defined CT criteria for the diagnosis of COVID-19. Nevertheless, given the moderate agreement among the three readers in our study, we can assert that chest CT is a reliable diagnostic method for use in the scenario of the COVID-19 pandemic.

As reported in other studies of the use of chest CT in COVID-19^(18-20)^, ground-glass opacities were the most common finding in our patient sample. According to Pan et al.^(14)^, ground-glass opacities are characteristic of the initial stage of the disease. However, they can also be found in the absorption stage, when the inflammatory process, characterized by consolidation, regresses^(21)^. It is essential to provide information on the time of disease progression in order to make a more accurate interpretation of the meaning of ground-glass opacities.

In addition to ground-glass opacities, other CT findings significantly associated with the diagnosis of COVID-19 were the crazy-paving pattern, consolidations, parenchymal bands, subpleural lines, nodules/consolidations with the halo sign, and vascular thickening. That is relevant because it suggests that there is a need to change the RSNA criteria for CT patterns in COVID-19. Case reports have cited nodules with the halo sign as typical CT findings in pediatric patients in the early stage of COVID-19^(22)^, which is in agreement with our finding that the halo sign was more common in patients with less than 50% parenchymal involvement. The diagnosis of vascular thickening is based on the finding of peripheral arterioles with a diameter > 3 mm^(23)^. This sign is better recognized when surrounded by ground-glass opacities^(24)^. In the more advanced stages of COVID-19, consolidations make it more challenging to recognize vascular thickening. After the regression of the inflammatory process, vessels return to a normal caliber. This phenomenon is associated with the set of inflammatory changes in COVID-19, and it originates from the imbalance of angiotensin II levels that causes neoangiogenesis or vasodilation^(24)^. Vascular thickening is an extremely relevant finding because of its high prevalence in COVID-19 and the fact that it is an uncommon finding in other types of pneumonia^(5,20)^. Bai et al.^(25)^ reported vascular thickening in 59% of patients with COVID-19, compared with only 22% of those with other types of pneumonia. The frequency of vascular thickening in our sample (22.9%) was lower than that reported in a study conducted by Caruso et al.^(26)^.

The need for mechanical ventilation is an indirect sign of disease severity. In a study of 5,700 patients hospitalized with COVID-19 in New York City, Richardson et al.^(27)^ showed that the mortality rate among patients requiring mechanical ventilation was 88.1%. In the present study, we found that parenchymal bands, bronchial ectasia, and peribronchovascular consolidations were more common in patients who required mechanical ventilation than in those who did not. Those CT signs are observed in cases of organizing pneumonia and are associated with the vascular phase of COVID-19, in which there is intra-alveolar fibrin and microthrombi, together with organized pneumonia and hyaline membranes^(28,29)^. In addition, we detected a positive association between the percentage of parenchymal involvement and the need for mechanical ventilation requirement. Therefore, we concluded that patients with more extensive signs of organizing pneumonia (parenchymal bands, bronchial ectasia, and peribronchovascular consolidations) were more likely to require ventilatory support. The relevance of these findings is supported by the results of autopsy studies of patients in whom the disease had progressed for more than 20 days, the main finding being acute fibrinous and organizing pneumonia^(30)^. Other relevant factors such as age and the presence of comorbidities were not directly associated with the need for mechanical ventilation in our patient sample.

The assessment of the extent of parenchymal involvement in our study is in keeping with previous studies that reported the importance of classifying the degree of parenchymal involvement, not only in terms of the extent but also in terms of the type of lesion in the lung, compared with the time of disease progression^(31)^, conventional and peribronchovascular consolidations being associated with more extensive parenchymal involvement. As in other studies^(18,32,33)^, we found that pleural effusion, despite being an uncommon finding in COVID-19, was associated with more extensive parenchymal involvement and therefore, theoretically, with greater disease severity.

In our sample, the patients with comorbidities had more extensive parenchymal involvement than did those without comorbidities. In addition, the least extensive parenchymal involvement (< 50%) was seen in the early stage of COVID-19 (when peripheral ground-glass nodules are typically predominant) and in the absorption stage (after day 14). We found that the parenchymal involvement was most extensive in the intermediate subcategories, which were the phases of transition to organizing pneumonia and the vascular phase. The same reasoning applies to the associations between proportional involvement and the Pan et al.^(14)^ classification: less extensive involvement (≤ 50%) occurred early, and the most extensive involvement (> 50%) occurred in the progression stage (stage 2) and in the peak stage (stage 3).

Our study has several limitations. First, there was no testing for other viruses. In addition, some clinical data were unavailable, because of the large number of outpatients who were discharged. Furthermore, most of the CT examinations were unenhanced and we therefore could not study the vascular complications of COVID-19.

In conclusion, the patients diagnosed with COVID-19 by CT were older than were those in whom the CT findings were negative for the disease. The main CT findings in the patients diagnosed with COVID-19 by CT were ground-glass opacities, consolidations (conventional or peribronchovascular), the crazy-paving pattern, parenchymal bands, vascular thickening, and nodules/consolidations with the halo sign. The typical and possible CT patterns for COVID-19, in the context of a pandemic, can be considered suggestive of the disease and agreed with the PCR results. To increase the specificity of CT, other signs should be included in the description of the typical pattern of COVID-19. Finally, patients in whom CT shows greater parenchymal involvement are more likely to require ventilatory support. Such patients typically present with signs suggestive of organizing pneumonia and have one or more comorbidities.

## Figures and Tables

**Table 1:** Characteristics of the patients diagnosed with COVID-19 by CT, by the need for mechanical ventilation and the extent of parenchymal involvement.

Variable	Positive CT (n = 105)	Mechanical ventilation required				Extent of involvement		
		Yes (n = 8)	No (n = 97)	*P*-value		< 50% (n = 59)	> 50% (n = 46)	*P*-value
Age, mean ± SD	54.8 ± 14.5	58.3 ± 14.8	54.5 ± 14.5	0.48*		54.0 ±14.3	55.8 ± 14.8	0.54
Pan et al.^**(**^^14^^**)**^ classification, n (%)								
Stage 1	32 (30.5)	0 (0.0)	32 (33.0)	0.14^†^		28 (47.5)	4 (8.7)	< 0.000^†^
Stage 2	41 (39.0)	4 (50.0)	37 (38.1)			19 (32.2)	22 (47.8)	
Stage 3	30 (28.6)	4 (50.0)	26 (26.8)			10 (16.9)	20 (43.5)	
Stage 4	2 (1.9)	0 (0.0)	2 (2.1)			2 (3.4)	0 (0.0)	
Time since symptom onset, n (%)								
1-4 days	27 (25.7)	1 (12.5)	26 (26.8)	0.80^‡^		21 (35.6)	6 (13.0)	0.011^‡^
5-8 days	43 (41.0)	4 (50.0)	39 (40.2)			18 (30.5)	25 (54.3)	
9-13 days	18 (17.1)	1 (12.5)	17 (17.5)			8 (13.6)	10 (21.7)	
> 14 days	17 (16.2)	2 (25.0)	15 (15.5)			12 (20.3)	5 (10.9)	
Comorbidities, n (%)								
Yes	50 (47.6)	6 (75.0)	44 (45.4)	0.10^†^		21 (35.6)	29 (63.0)	0.005^†^
No	55 (52.4)	2 (25.0)	53 (54.6)			38 (64.4)	17 (37.0)	
Extent of involvement, n (%)								
0-25%	37 (35.2)	0 (0.0)	37 (38.1)	0.023^†^				
25-50%	22 (21.0)	1 (12.5)	21 (21.6)					
> 50%	46 (43.8)	7 (87.5)	39 (40.2)					

* Student's t-test for independent samples.

† Fisher's exact test.

‡ Mann–Whitney test.

**Table 2:** Distribution of CT findings and their associations with the need for mechanical ventilation and the extent of parenchymal involvement.

CT finding	Positive CT (n = 105) n (%)	Mechanical ventilation required				Extent of involvement		
		Yes (n = 8) n (%)	No (n = 97) n (%)	*P*-value*		< 50% (n = 59) n (%)	> 50% (n = 46) n (%)	*P*-value*
Ground-glass opacity	93 (88.6)	7 (87.5)	86 (88.7)	0.63		53 (89.8)	40 (87.0)	0.65
Crazy-paving	44 (41.9)	4 (50.0)	40 (41.2)	0.45		21 (35.6)	23 (50.0)	0.14
Parenchymal bands	34 (32.4)	6 (75.0)	28 (28.9)	0.013		15 (25.4)	19 (41.3)	0.084
Subpleural lines	15 (14.3)	0 (0)	15 (15.5)	0.28		10 (16.9)	5 (10.9)	0.38
Conventional consolidations	45 (42.9)	6 (75.0)	39 (40.2)	0.062		18 (30.5)	27 (58.7)	0.003
Bronchial ectasia	12 (11.4)	3 (37.5)	9 (9.3)	0.046		4 (6.8)	8 (17.4)	0.090
Architectural distortion	7 (6.7)	1 (12.5)	6 (6.2)	0.44		2 (3.4)	5 (10.9)	0.13
Peribronchial/vascular consolidations	23 (21.9)	5 (62.5)	18 (18.6)	0.012		8 (13.6)	15 (32.6)	0.019
Nodule/consolidations with the halo sign	15 (14.3)	0 (0)	15 (15.5)	0.28		14 (23.7)	1 (2.2)	0.001
Reversed halo sign	1 (1.0)	0 (0)	1 (1.0)	0.92		1 (1.7)	0 (0)	0.56
Vascular thickening	24 (22.9)	2 (25.0)	22 (22.7)	0.59		10 (16.9)	14 (30.4)	0.10
Bubble sign	3 (2.9)	0 (0)	3 (3.1)	0.79		1 (1.7)	2 (4.3)	0.41
Pleural effusion	10 (9.5)	1 (12.5)	9 (9.3)	0.56		2 (3.4)	8 (17.4)	0.017

* Chi-square test or Fisher's exact test.
